# The cognitive defects of neonatally irradiated mice are accompanied by changed synaptic plasticity, adult neurogenesis and neuroinflammation

**DOI:** 10.1186/1750-1326-9-57

**Published:** 2014-12-16

**Authors:** Stefan J Kempf, Arianna Casciati, Sonja Buratovic, Dirk Janik, Christine von Toerne, Marius Ueffing, Frauke Neff, Simone Moertl, Bo Stenerlöw, Anna Saran, Michael J Atkinson, Per Eriksson, Simonetta Pazzaglia, Soile Tapio

**Affiliations:** Institute of Radiation Biology, Helmholtz Zentrum München, German Research Center for Environmental Health, 85764 Neuherberg, Germany; Laboratory of Radiation Biology and Biomedicine, Agenzia Nazionale per le Nuove Tecnologie, l’Energia e lo Sviluppo Economico Sostenibile (ENEA) Centro Ricerche (CR) Casaccia, 00123 Rome, Italy; Department of Environmental Toxicology, Uppsala University, 75236 Uppsala, Sweden; Institute of Pathology, Helmholtz Zentrum München, German Research Center for Environmental Health, 85764 Neuherberg, Germany; Research Unit Protein Science, Helmholtz Zentrum München, German Research Center for Environmental Health, 85764 Neuherberg, Germany; Division of Biomedical Radiation Sciences, Svedberg Laboratory, Uppsala University, 75105 Uppsala, Sweden; Chair of Radiation Biology, Technische Universität München, 81675 Munich, Germany

**Keywords:** Dendritic spines, Hippocampus, Cortex, CREB, miR-132, Ionising radiation, Proteomics, Rac1, Cofilin, Alzheimer

## Abstract

**Background/purpose of the study:**

Epidemiological evidence suggests that low doses of ionising radiation (≤1.0 Gy) produce persistent alterations in cognition if the exposure occurs at a young age. The mechanisms underlying such alterations are unknown. We investigated the long-term effects of low doses of total body gamma radiation on neonatally exposed NMRI mice on the molecular and cellular level to elucidate neurodegeneration.

**Results:**

Significant alterations in spontaneous behaviour were observed at 2 and 4 months following a single 0.5 or 1.0 Gy exposure. Alterations in the brain proteome, transcriptome, and several miRNAs were analysed 6–7 months post-irradiation in the hippocampus, dentate gyrus (DG) and cortex. Signalling pathways related to synaptic actin remodelling such as the Rac1-Cofilin pathway were altered in the cortex and hippocampus. Further, synaptic proteins MAP-2 and PSD-95 were increased in the DG and hippocampus (1.0 Gy). The expression of synaptic plasticity genes Arc, c-Fos and CREB was persistently reduced at 1.0 Gy in the hippocampus and cortex. These changes were coupled to epigenetic modulation via increased levels of microRNAs (miR-132/miR-212, miR-134). Astrogliosis, activation of insulin-growth factor/insulin signalling and increased level of microglial cytokine TNFα indicated radiation-induced neuroinflammation. In addition, adult neurogenesis within the DG was persistently negatively affected after irradiation, particularly at 1.0 Gy.

**Conclusion:**

These data suggest that neurocognitive disorders may be induced in adults when exposed at a young age to low and moderate cranial doses of radiation. This raises concerns about radiation safety standards and regulatory practices.

**Electronic supplementary material:**

The online version of this article (doi:10.1186/1750-1326-9-57) contains supplementary material, which is available to authorized users.

## Background

Ionising radiation remains a first-line treatment for malignancies of the central nervous system (CNS). However, therapeutic doses of cranial irradiation can lead to a long-lasting decrease in cognition and visual memory [1], the severity of which is more pronounced in children younger than three years at the time of treatment [2, 3]. Epidemiological evidence suggests that doses considerably lower than those used in radiotherapy may also lead to cognitive impairment in the young [4–6], possibly due to the extensive remodelling of the immature brain.

Indeed, toxins are more effective in disrupting adult brain function in mice alone or in combination with irradiation if the exposure occurs around neonatal day ten [7, 8]. A possible mechanism for the detriment is indicated by the persistent alteration of hippocampal processes by high radiation doses. This is associated with a depletion of stem and progenitor cells within the neurogenic niche of the hippocampal dentate gyrus (DG) [9]. Alternatively, changes may occur at the level of mature neuronal networks. Alterations in neural dendrite and spine morphology are in fact reported after irradiation [10], similar to those observed in cognitive disorders such as Alzheimer’s [11], Rett syndrome [12] and Down’s syndrome [13].

We have previously shown that ionising radiation immediately impairs synaptic plasticity-associated signalling pathways both in murine hippocampal neuronal HT22 cells and in mouse brain [14]. The aim of this study was to investigate long-term effects of low and moderate doses of irradiation on cognition and to elucidate the molecular mechanisms behind the possible damage. For this purpose, male NMRI mice were exposed to a single dose of total body gamma irradiation of 0 (sham), 0.02, 0.1, 0.5 and 1.0 Gy on postnatal day 10 (PND 10). Doses as low as 0.5 Gy had persistent effects on cognitive behaviour associated with alterations in Rac1-Cofilin pathway, increased neuroinflammation and decrease in adult neurogenesis.

## Results

### Ionising radiation induces a reduction in cognitive performance

**Figure 1:**
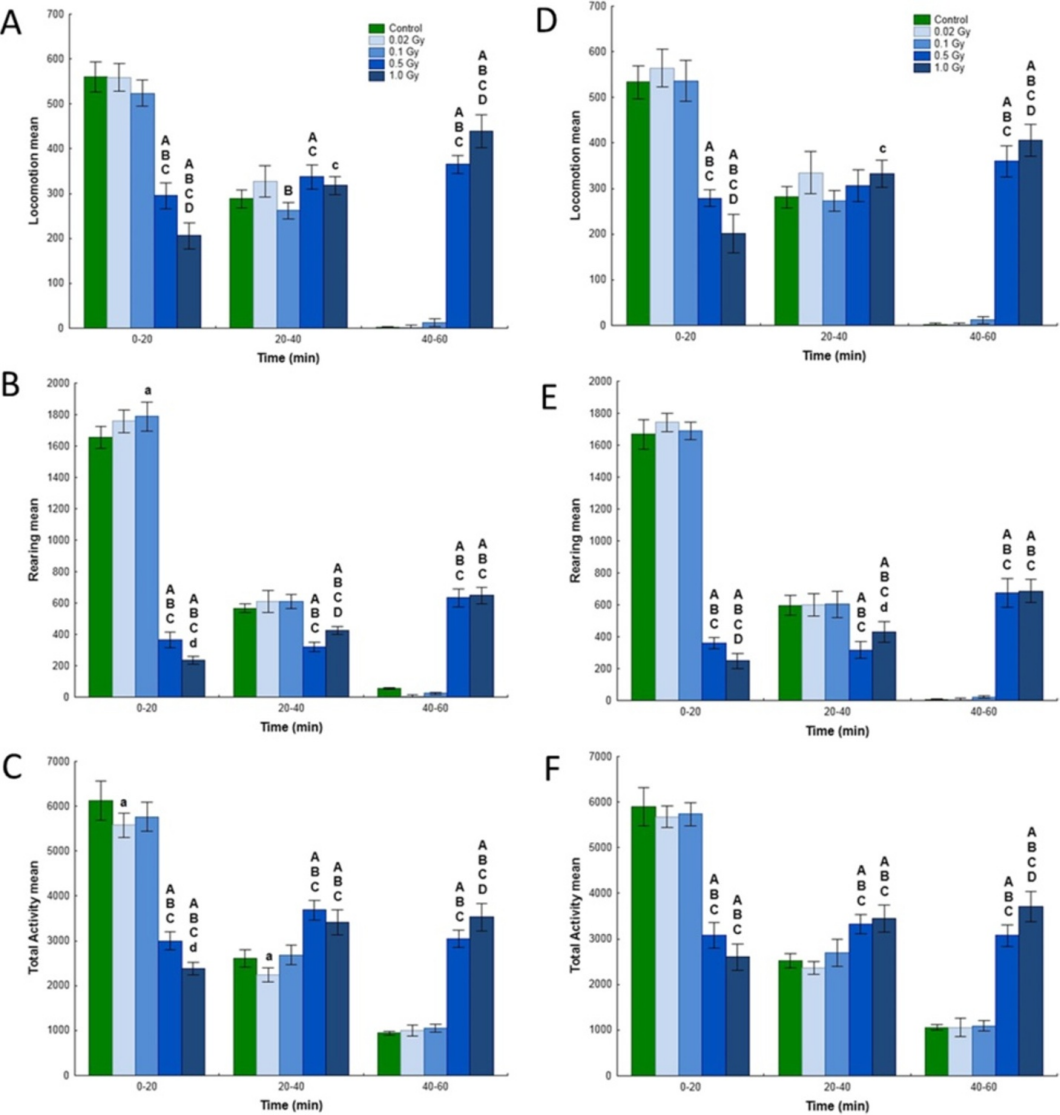
**Analysis of spontaneous behaviour.** Spontaneous behaviour of 2-month-old (**A**, **B** and **C**) and 4-month-old (**D**, **E** and **F**) NMRI male mice were exposed to 0, 0.02, 0.1, 0.5 or 1.0 Gy gamma radiation on postnatal day 10. The data were subjected to an ANOVA with split-plot design and significant treatment × time interactions were observed for 2-month-old and 4-month-old behaviour including locomotion, rearing, and total activity. Pairwise testing between control animals and animals exposed to gamma radiation was performed using Duncan’s MRT test. The statistical differences are indicated as: **(A)** significantly different vs. control, p ≤ 0.01; (a) significantly different vs. control, p ≤ 0.05; **(B)** significantly different vs. 0.02 Gy, p ≤ 0.01; (b) significantly different vs. 0.02 Gy, p ≤ 0.05; **(C)** significantly different vs. 0.1 Gy, p ≤ 0.01; (c) significantly different vs. 0.1 Gy, p ≤ 0.05; **(D)** significantly different vs. 0.5 Gy, p ≤ 0.01; (d) significantly different vs. 0.5 Gy, p ≤ 0.05; n = 12 per exposure group.

Spontaneous behaviour in a novel home environment revealed significant radiation-induced alterations 2 and 4 months post-irradiation (Figure 1). Two months after exposure there were significant treatment × time interactions for locomotion [F(8,110) = 159.90], rearing [F(8,110) = 404.05] and total activity [F(8,110) = 145.14]. Four months after exposure significant treatment × time interactions remained for all three variables, locomotion [F(8,110) = 100.43], rearing [F(8,110) = 328.03] and total activity [F(8,110) = 111.18]. Pair-wise tests between irradiated and sham-irradiated groups showed significant dose-related changes in all three variables. Behavioural changes were seen in mice exposed to 0.5 and 1.0 Gy but not at lower doses.

### Persistent radiation-induced changes in actin cytoskeleton-associated signalling pathways in hippocampus and cortex

**Figure 2:**
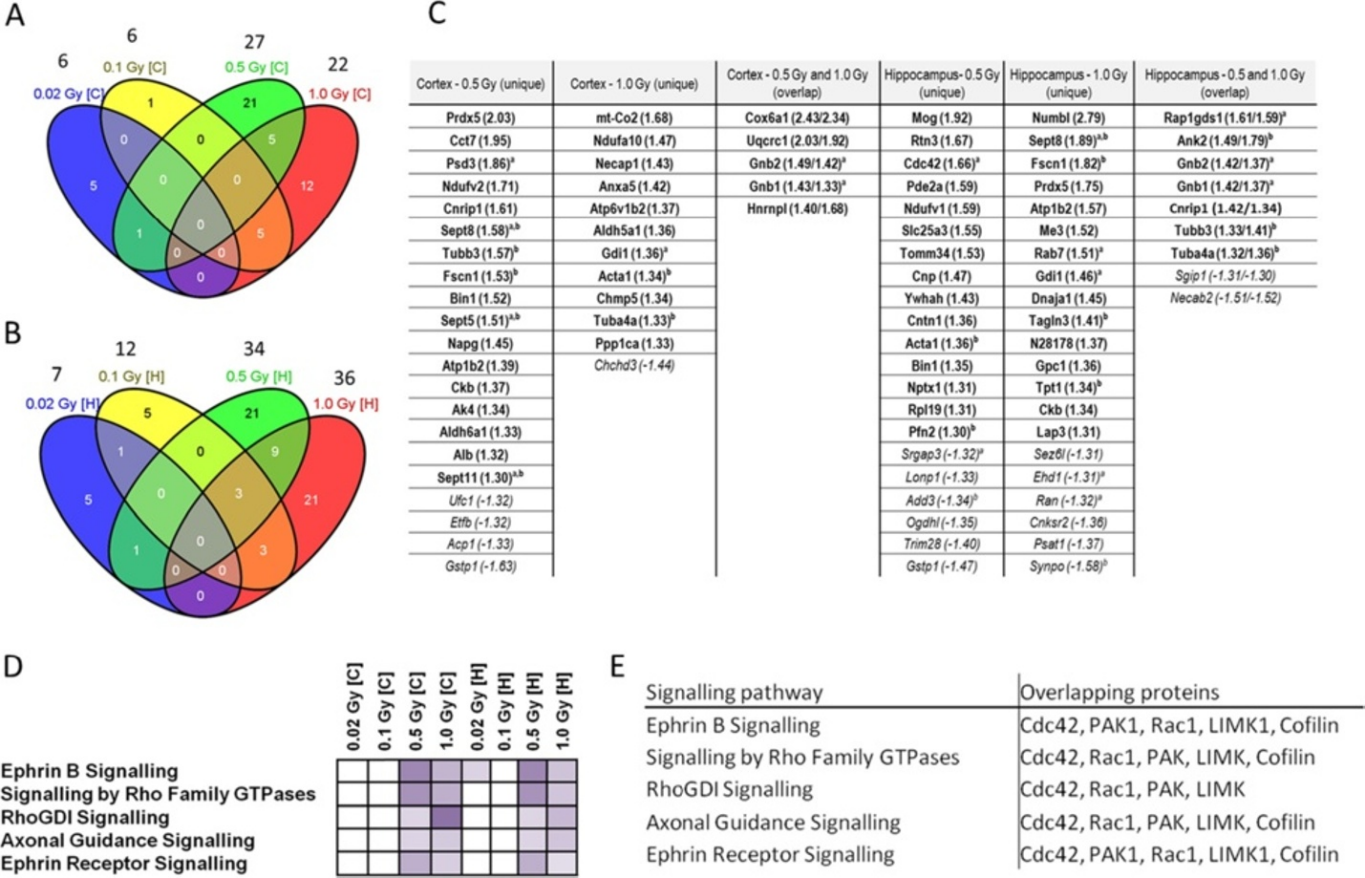
**Analysis of signalling pathways from proteomic experiments.** Venn diagrams of deregulated proteins from cortex [C] **(A)** and hippocampus [H] **(B)** exposed to 0.02 Gy, 0.1 Gy, 0.5 Gy and 1.0 Gy from global proteomics approach are shown. H: n = 4; C: n = 5. The number above each dose shows the total number of deregulated proteins at this dose. The unique and overlapping significantly deregulated proteins in hippocampus and cortex at doses of 0.5 Gy and 1.0 Gy are shown in panel **C**. Each column shows the proteins significantly up-regulated (bold) or down-regulated (italic) with the fold-changes in brackets. Proteins indicated with a and/or b belong to the protein class of “small GTPase/associated G-protein” and/or “cytoskeleton / cytoskeleton-binding protein”, respectively, as categorised using the PANTHER software tool and UniProt database. Hippocampal and cortical data result from four and five biological replicates. Associated signalling pathways of all dose-dependent significantly deregulated proteins using the Ingenuity Pathway Analysis (IPA) software are shown in panel **D**. Higher colour intensity represents higher significance (p-value) whereas all coloured boxes have a p-value of ≤ 0.05; white boxes have p-value of > 0.05 and are not significant. Hippocampal and cortical data result from four and five biological replicates, respectively. H: Hippocampus, C: Cortex. Panel **E** shows the overlapping proteins within the in panel D depicted signalling pathways.

To identify the potential mechanisms of cognitive alterations, we quantified protein changes in hippocampus and cortex seven months post-irradiation. The number of significantly deregulated proteins was increased in a dose-dependent manner (cortex/hippocampus: 0.02 Gy – 6/7 proteins, 0.1 Gy – 6/12 proteins, 0.5 Gy – 27/34 proteins, and 1.0 Gy – 22/36 proteins) (Figure 2A – B, Additional file 1: Table S6). The complete list of quantifiable proteins is shown in Additional file 1: Table S4. In agreement with the behavioural changes seen at 0.5 and 1.0 Gy, the deregulated proteins at these doses were partly overlapping and distinct from the deregulated proteins seen at the lower doses (Figure 2A - B). Gene ontology (GO) analysis revealed a number of altered proteins unique at the higher doses to be small GTPases, GTPase-associated proteins or cytoskeletal proteins (Figure 2C), or to be involved in signalling via ephrin B- ephrin receptor, Rho family GTPases, RhoGDI or axonal guidance signalling (Figure 2D). The shared deregulated proteins of these pathways included the small GTPases Rac1 and Cdc42, the kinases PAK and LIMK1, and the actin-modulating cofilin (Figure 2E); all these proteins are functionally associated to axonal maturation, spine and synapse formation, maturation and morphology via regulation of actin polymerisation of the Rac1-Cofilin pathway [15, 16].

### Ionising radiation impacts the Rac1-Cofilin signalling pathway

**Figure 3:**
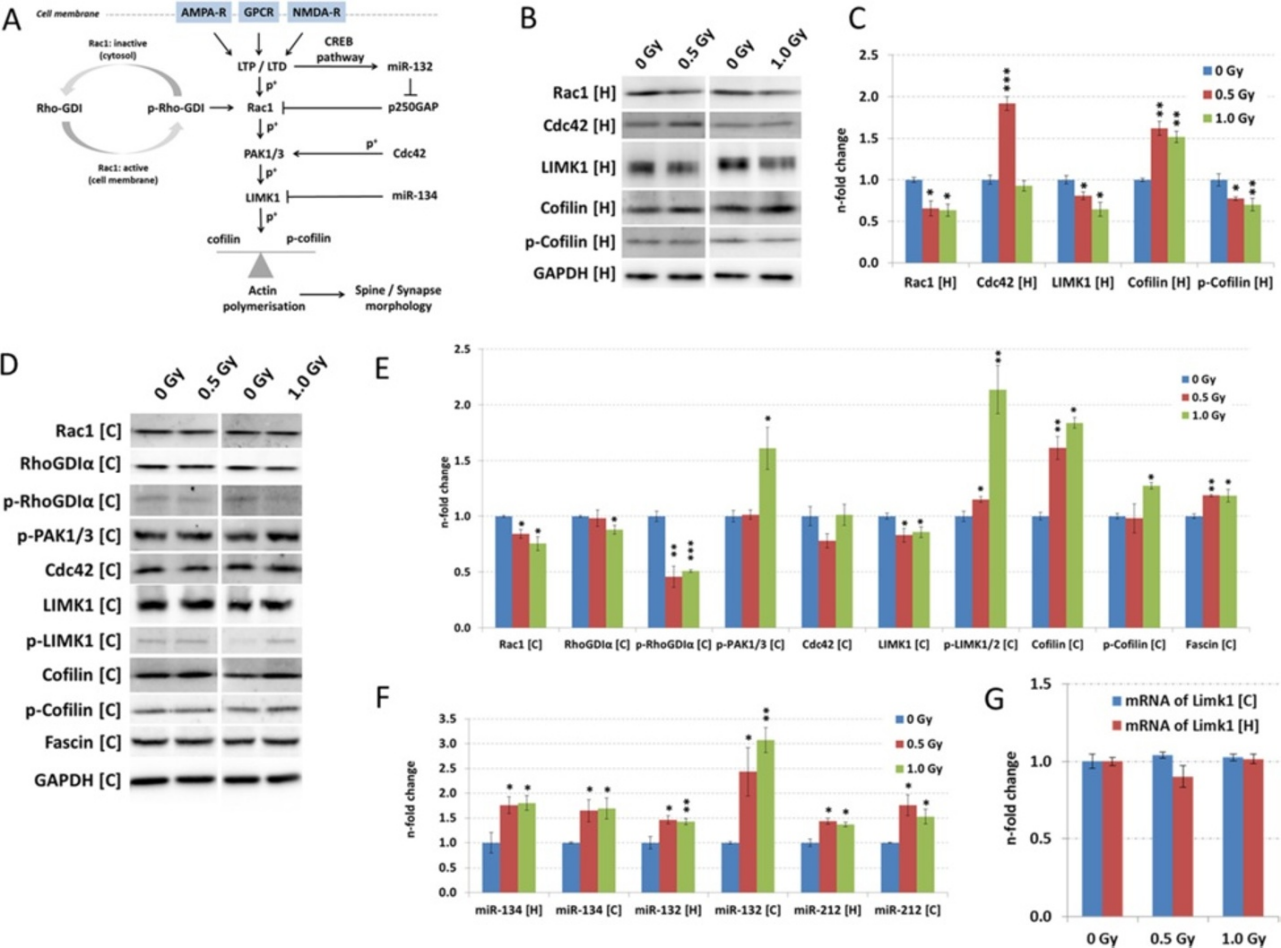
**The Rac1-Cofilin signalling pathway and subsequent analysis of involved molecules.** The Rac1-Cofilin signalling pathway is shown **(A)**. Signalling from AMPA, NMDA and GPC receptors leads to activation (phosphorylation) of Rac1 via LTP / LTD. This leads to downstream activation (phosphorylation) of PAK1/3, LIMK1 and final inactivation of cofilin via phosphorylation. Cdc42 is also able to phosphorylate PAK1/3. Associated microRNAs are miR-132 regulating Rac1 activity via modulation of a GTP hydrolysis protein (p250GAP) and miR-134 directly suppressing LIMK1 levels. Rac1 activity is regulated via phosphorylation of RhoGDI releasing Rac1 from RhoGDI inhibitory complex. Data from immunoblots (**B - E**) and quantification of miRNAs associated with the Rac1-Cofilin pathway **(F)** are shown in hippocampus and cortex from control, and exposed mice (0.5 and 1.0 Gy). The columns represent the fold-changes with standard errors of the mean (SEM) from three biological replicates. The visualisation of protein bands shows the representative change from three biological replicates. *p < 0.05; **p < 0.01; ***p < 0.001 (unpaired Student’s t-test). Normalisation was performed against endogenous GAPDH and endogenous snoRNA135 for immunoblotting and miRNA quantification, respectively. Gene expression analysis of Limk1 in cortex [C] and hippocampus [H] exposed to sham-irradiation, 0.5 Gy and 1.0 Gy is shown in panel **G**. Columns represent fold-changes with the standard error of the mean (SEM) from 3 biological replicates. Statistical analysis was performed with unpaired Student’s t-test; H: Hippocampus, C: Cortex.

Using immunoblotting and miRNA quantification, we observed defects in the Rac1-Cofilin pathway (Figure 3A). Irradiation with doses of 0.5 Gy and 1.0 Gy significantly decreased Rac1 and LIMK1 protein expression both in the cortex and hippocampus (Figure 3B – E). Cofilin, a target for negative regulation by Rac1, was elevated in both brain regions at 0.5 Gy and 1.0 Gy (Figure 3B – E). Simultaneously, inactive (phosphorylated) cofilin was increased in the cortex at the highest dose (1.0 Gy) but decreased in hippocampus at both 0.5 and 1.0 Gy (Figure 3B – E). Further, quantification of Cdc42 demonstrated an increase only at 0.5 Gy in the hippocampus (Figure 3B - C) correlating with proteomics data (Figure 2C) whereas Cdc42 protein levels were not changed in the cortex (Figure 3D - E).

Other components of the Rac1-Cofilin pathway were also influenced. The level of RhoGDIα in the cortex was slightly decreased at 1.0 Gy but unchanged at 0.5 Gy whereas the phosphorylated (inactive state to sequester Rac1) form was strongly decreased at both doses (Figure 3D - E). Cortical levels of phosphorylated (active) PAK1/3 and phosphorylated (active) LIMK1/2 were increased only at 1.0 Gy (Figure 3D - E). This is in good agreement with the increased levels of phosphorylated cofilin at this dose (Figure 3D - E).

Fascin plays an important role in the organisation of actin filament bundles whereas cofilin may play a cooperative role in disassembly of filopodial actin filaments as demonstrated *in vitro* [17]. Immunoblotting analysis demonstrated a significant increase in cortical fascin levels at both doses (Figure 3D - E), in accordance with proteomics data of fascin1 (Fscn1) levels at 0.5 Gy (Figure 2C).

As LIMK1 protein levels were down-regulated without related changes in gene expression (Figure 3G) in cortex and hippocampus, we measured the level of miR-134, a negative regulator of LIMK1. The decrease of LIMK1 protein was coinciding with a significant increase in miR-134 levels both in cortex and hippocampus (Figure 3F). We also evaluated the levels of miR-132 and its tandem miRNA miR-212; they were both significantly increased in hippocampus and cortex (Figure 3F).

### Ionising radiation induces changes in synaptic proteins in the hippocampus

**Figure 4:**
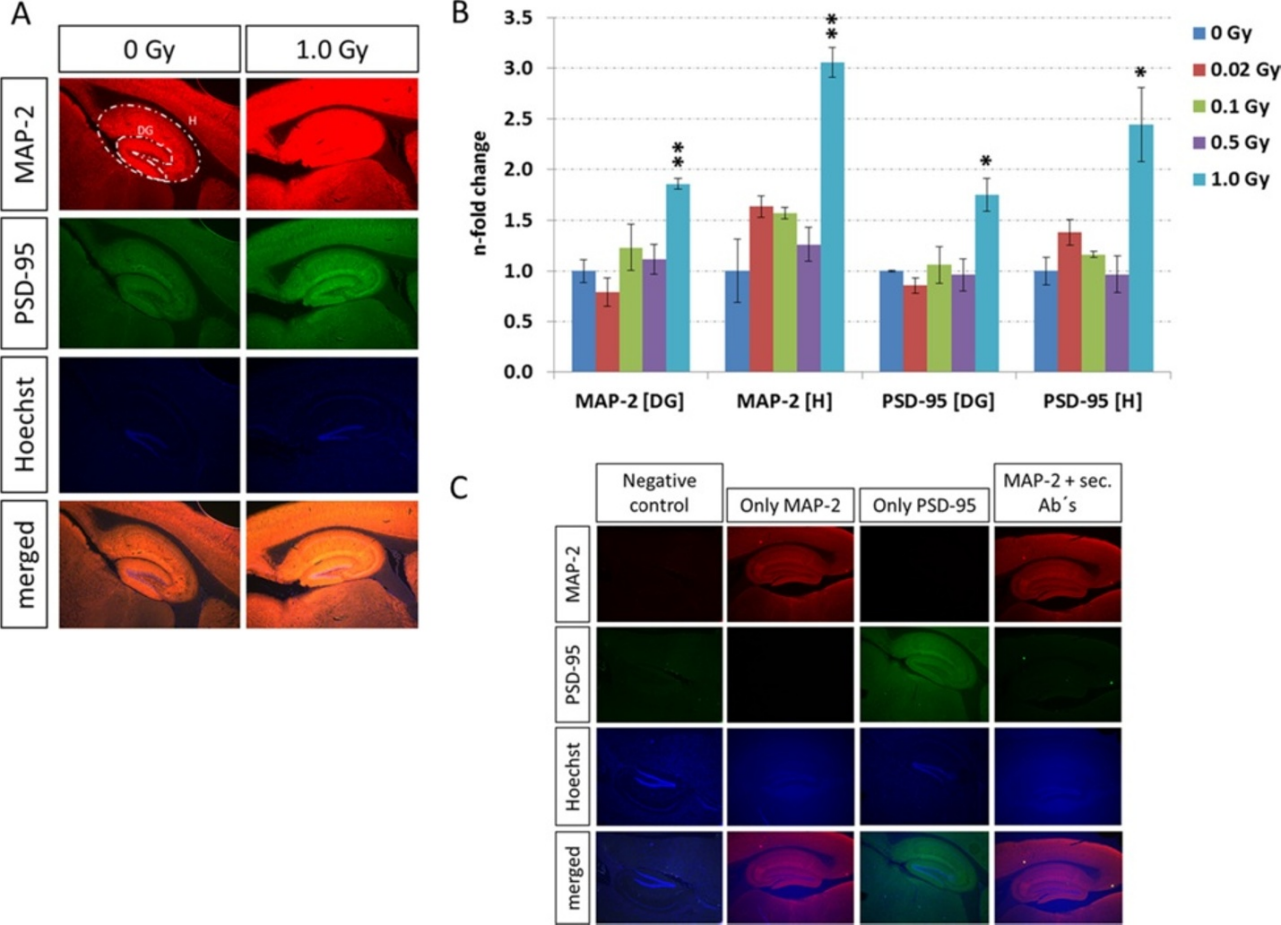
**Analysis of synaptic proteins via sequential immunofluorescence in hippocampus and dentate gyrus.** Data from sequential immunofluorescence from hippocampus (H) and dentate gyrus (DG) at different doses are shown (**A** and **B**). The columns represent the fold-changes with standard errors of the mean (SEM) from three biological replicates. The visualisation shows the representative intensity from three biological replicates of 0 Gy and 1.0 Gy regarding MAP-2 (red - Microtubule-associated protein 2), PSD-95 (green - Disks large homolog 4 (DLG4), Hoechst (blue) and merged intensities within the hippocampal region. The MAP-2 / PSD-95 intensity was normalised against nuclear Hoechst intensity in the region of interest. *p < 0.05; **p < 0.01; ***p < 0.001 (unpaired Student’s t-test); magnification: 4x. Representative images of sequential immunofluorescence from hippocampus are shown in panel **C**. Images indicate the specific binding of secondary antibodies (“negative control”), binding sites of primary MAP-2 antibody saturated via Cy3-Fab-fragment IgG (“MAP-2 + sec. Ab’s”) and sequential immunofluorescence with a single protein detection (“Only MAP-2” and “Only PSD-95”). “Negative control” – only secondary antibodies and Hoechst; “only MAP-2” – primary antibody against MAP-2, Cy3-Fab-fragment IgG secondary antibody, Hoechst; “only PSD-95” – primary antibody against PSD-95, Alexa-fluor IgG secondary antibody, Hoechst; “MAP-2 + sec. Ab’s” – primary antibody against MAP-2, Cy3-Fab-fragment IgG secondary antibody, Alexa-fluor IgG secondary antibody, Hoechst; magnification: 4x.

To further investigate radiation-induced changes in synaptic processes the levels of the postsynaptic density protein 95 (PSD-95) and the microtubule-associated protein 2 (MAP-2) were measured by sequential immunofluorescence. Previous studies have shown their involvement in spine formation, stability and maturation and their localisation in neuronal dendrites [18, 19]. We noted significant increases of PSD-95 and MAP-2 only at 1.0 Gy in the hippocampus and DG (Figure 4A - B); respective controls are shown in Figure 4C.

### Radiation-induced defects in long-term potentiation/long-term depression signalling pathways are associated with imbalances in CREB-dependent neuronal activity

**Figure 5:**
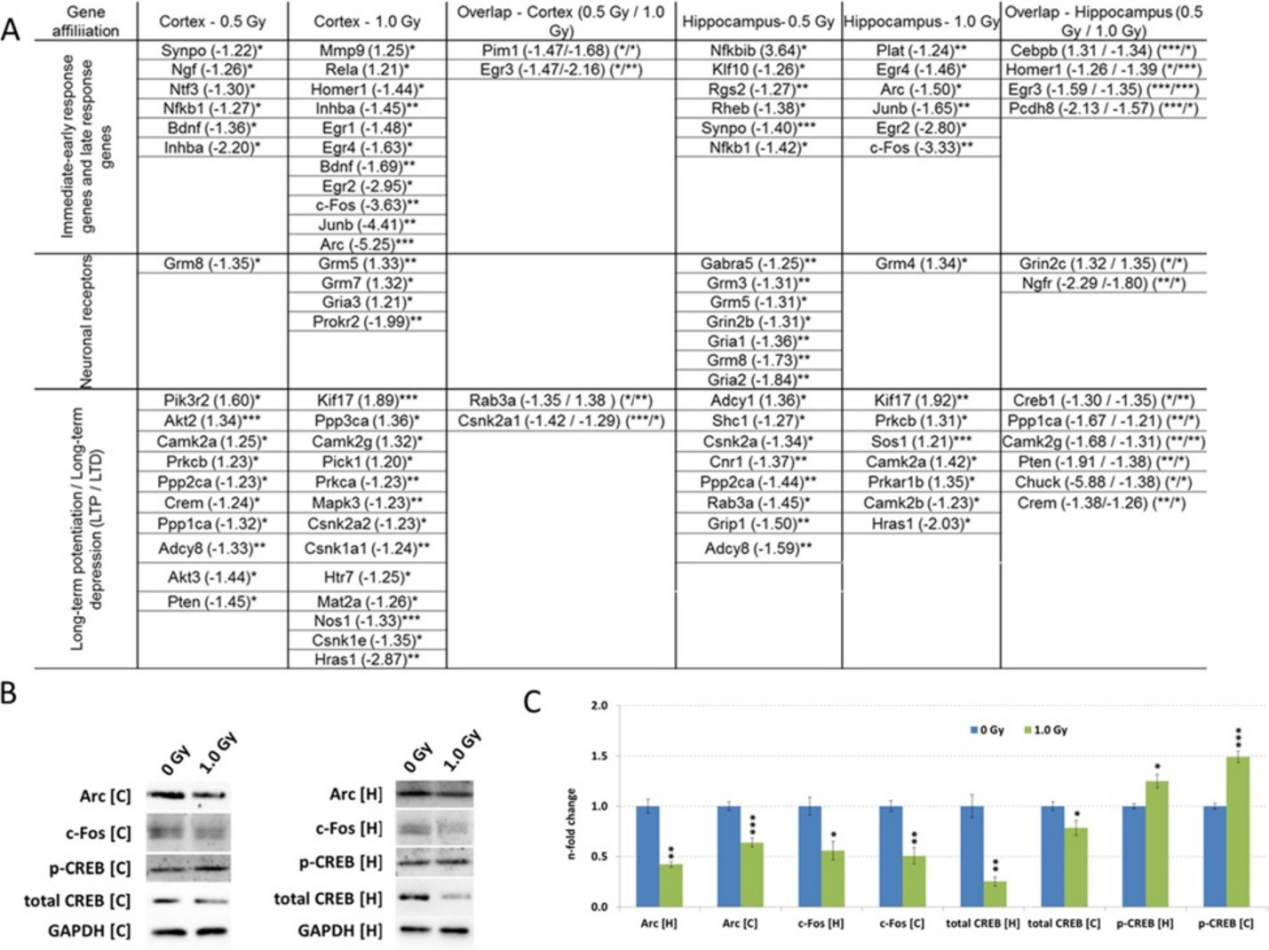
**Quantification of the expression of genes and proteins involved in synaptic plasticity.** Genes significantly changed in expression from hippocampus and cortex at doses of 0.5 Gy and 1.0 Gy using RT2 Profiler PCR Arrays are shown in **A**. The table shows the genes which are significantly up-regulated or down-regulated with the fold-changes in brackets; *p < 0.05; **p < 0.01; ***p < 0.001 (unpaired Student’s t-test, n = 3) after normalisation to the median of 84 target genes. Immunoblots and quantification of Arc, c-Fos, total CREB and phosphorylated CREB in hippocampus and cortex of sham-irradiated and 1.0 Gy irradiated mice 7 months post irradiation (**B** and **C**). The columns represent the fold-changes with standard errors of the mean (SEM) from three biological replicates. The visualisation of protein bands shows the representative change using three biological replicates. *p < 0.05; **p < 0.01; ***p < 0.001 (unpaired Student’s t-test). Normalisation was performed against endogenous GAPDH; H: Hippocampus, C: Cortex.

Changes in synaptic plasticity could contribute to the observed behavioral impairments**.** To investigate gene expression related to synaptic plasticity, we quantified the expression levels of immediate-early response genes (IEG’s) as an indicator for neuronal activity immediately after neurotransmission. The expression of several of these genes was down-regulated in the hippocampus and cortex (Figure 5A, Additional file 1: Table S5). We confirmed that the observed marked decrease in the gene expression of *Arc* and *c-Fos* was paralleled by decrease of their protein expression in irradiated hippocampus and cortex (Figure 5B and C).

Changes in both long-term potentiation and -depression (LTP / LTD) are involved in physical modulation in synapse recycling and thus in neuronal receptors. Gene expression quantification of a panel of 21 receptors showed that several of these genes were decreased in their expression consisting of predominantly G-protein coupled receptors for glutamate (*Grm’s*) but also α-amino-3-hydroxy-5-methyl-4-isoxazolepropionic acid (AMPA) receptor-selective glutamate receptors (*Gria’s*) and N-methyl-D-aspartate (NMDA) receptors (*Grin’s*) in hippocampus and cortex (Figure 5A, Additional file 1: Table S5).

Based on these changes in the receptor profile, we quantified the downstream signalling pathways involved in LTP/LTD with the emphasis on the activation of the nuclear transcription factors CREB (cAMP responsive element binding protein) and CREM (cAMP response element modulator). Gene expression analysis showed alterations in modulators and upstream regulators of CREB such as protein kinases (*Prk’s*), adenylate cyclases (*Adcy’s*), serine/threonine-protein kinases (*Akt’s)*, calmodulin kinases (*Camk’s*), and casein kinases (*Csnk’s*) in both hippocampus and cortex (Figure 5A, Additional file 1: Table S5). Additionally, we observed decreased *Creb1* expression in hippocampus and decreased *Crem* levels in the cortex and hippocampus (Figure 5A). As there are several forms of the CREB protein, we quantified both total CREB and phosphorylated (active) CREB (Ser133) levels. A significantly reduced expression of total CREB accompanied with a slight increase in phosphorylated CREB was found in both hippocampus and cortex at 1.0 Gy (Figure 5B and C).

### Irradiation impairs adult neurogenesis in the hippocampus

**Figure 6:**
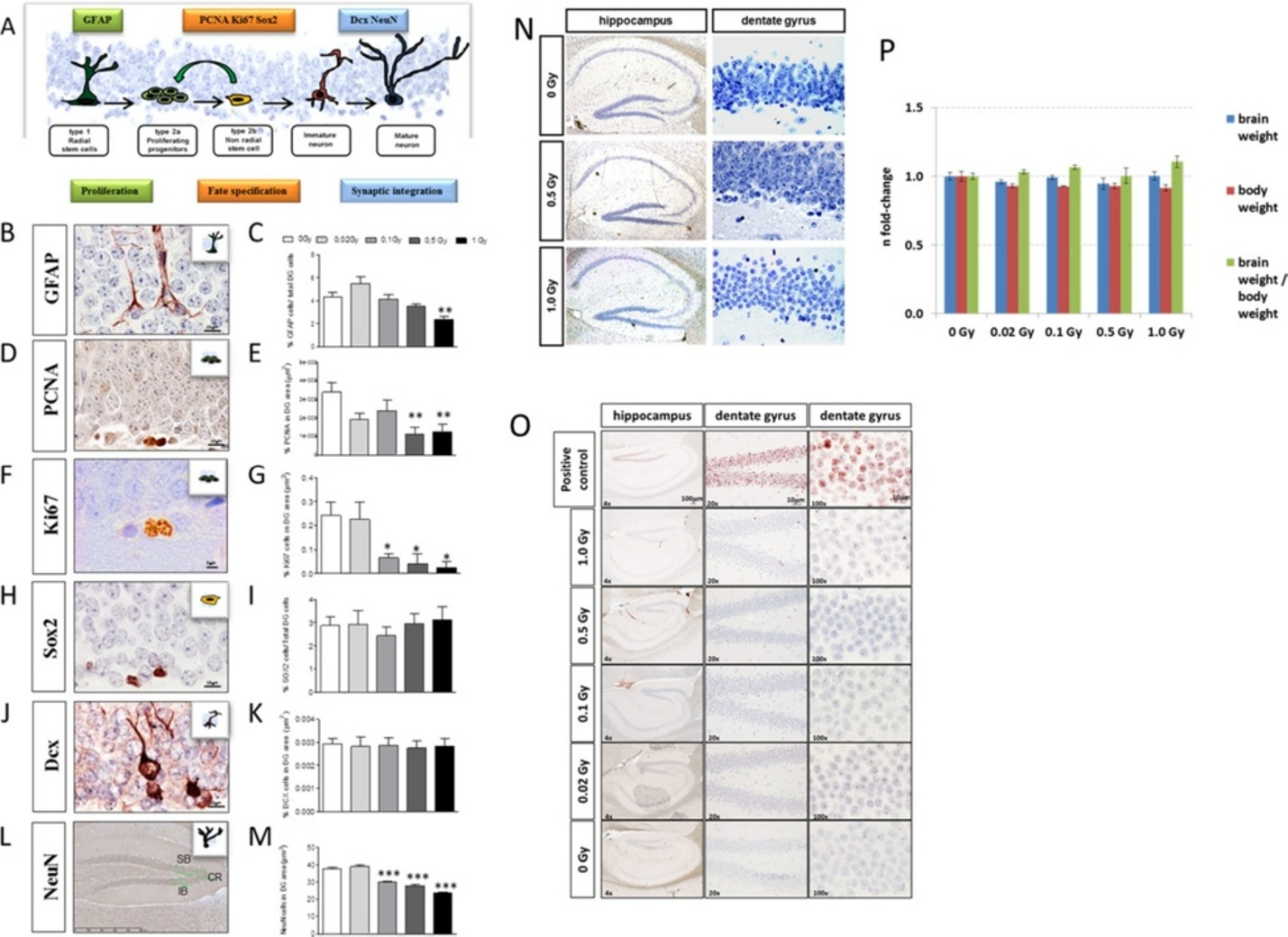
**Adult neurogenesis and evaluation of apoptosis, DNA strands and derivations in animal and brain weights.** Schematic representation of proneural markers for staging adult neurogenesis in the hippocampus by immunohistochemistry is shown **(A)**. Representative image of stem-like cells (type 1 radial glial) labelled by GFAP **(B)**, of proliferating progenitor cells (type 2a) labelled with PCNA **(D)** and Ki67 **(F)**, of non-radial stem cells (type 2b) labeled by Sox2 **(H)**, of newborn and mature neurons labeled by Dcx **(J)** and NeuN **(L)** and corresponding relative quantifications (**C**, **E**, **G**, **I**, **K**, **M**) are shown. NeuN+ cells were counted in 4000 μm^2^ areas (in green). *p < 0.05; **p < 0.01; ***p < 0.001 (unpaired Student’s t-test). Data are reported as mean ± SEM (n = 3 for Ki67, n ≥ 3 for NeuN, others n = 6); DG, dentate gyrus; CR, crest area; SB, suprapyramidal blade; IB, infrapyramidal blade. Visualisation of activated Caspase-3 **(N)** and γH2AX **(O)** in hippocampus and DG and fold-changes of brain weight, body weight and their ratios **(P)** are shown. The positive control of γH2AX consisted of hippocampus irradiated with 3.0 Gy and stained after 30 minutes. Data of weights are reported as fold-changes ± SEM from at least 5 biological replicates (Student’s t-test, unpaired).

Alterations in the cellular composition and kinetics of the subgranular and granular zones of the DG were evident from morphological and immunohistochemical analysis of stage-specific adult neurogenesis markers (Figure 6A). GFAP^+^ cells, with a radial glial-like morphology, represent the most primitive stem-like population in the DG. Mice irradiated with 1.0 Gy exhibited a significant decrease (46.2%) in the number of GFAP^+^-type 1 radial stem cells in the subgranular zone (SGZ) (Figure 6B - C). Further, we noted a dose-dependent decrease in type 2a proliferating progenitors labelled by PCNA (62 and 67%) at all doses ≥0.5 Gy and by Ki67 (73, 83 and 90%) at all doses ≥ 0.1 Gy (Figure 6D - G). Neither the percentage of small round/oval cells labelled by Sox2 (Figure 6H - I), nor the number of post-mitotic new-born neurons labelled by Dcx were modified by irradiation (Figure 6J - K). However, a dose-dependent decrease was observed in the number of mature neurons labelled by NeuN in the crest (CR), suprapyramidal- (SB) and infrapyramidal-blade (IB) regions of the DG at 0.1 Gy (21%), 0.5 Gy (26%) and 1.0 Gy (37%) (Figure 6L - M). Changes were not associated with either persistent DNA damage or apoptosis as neither γH2AX DNA damage foci nor cleaved caspase-3 were detected (Figure 6N - O). The observed cell losses were not generalised as brain/body weight ratios were unaffected by radiation history (Figure 6P).

### Radiation exposure induces neuroinflammation

**Figure 7:**
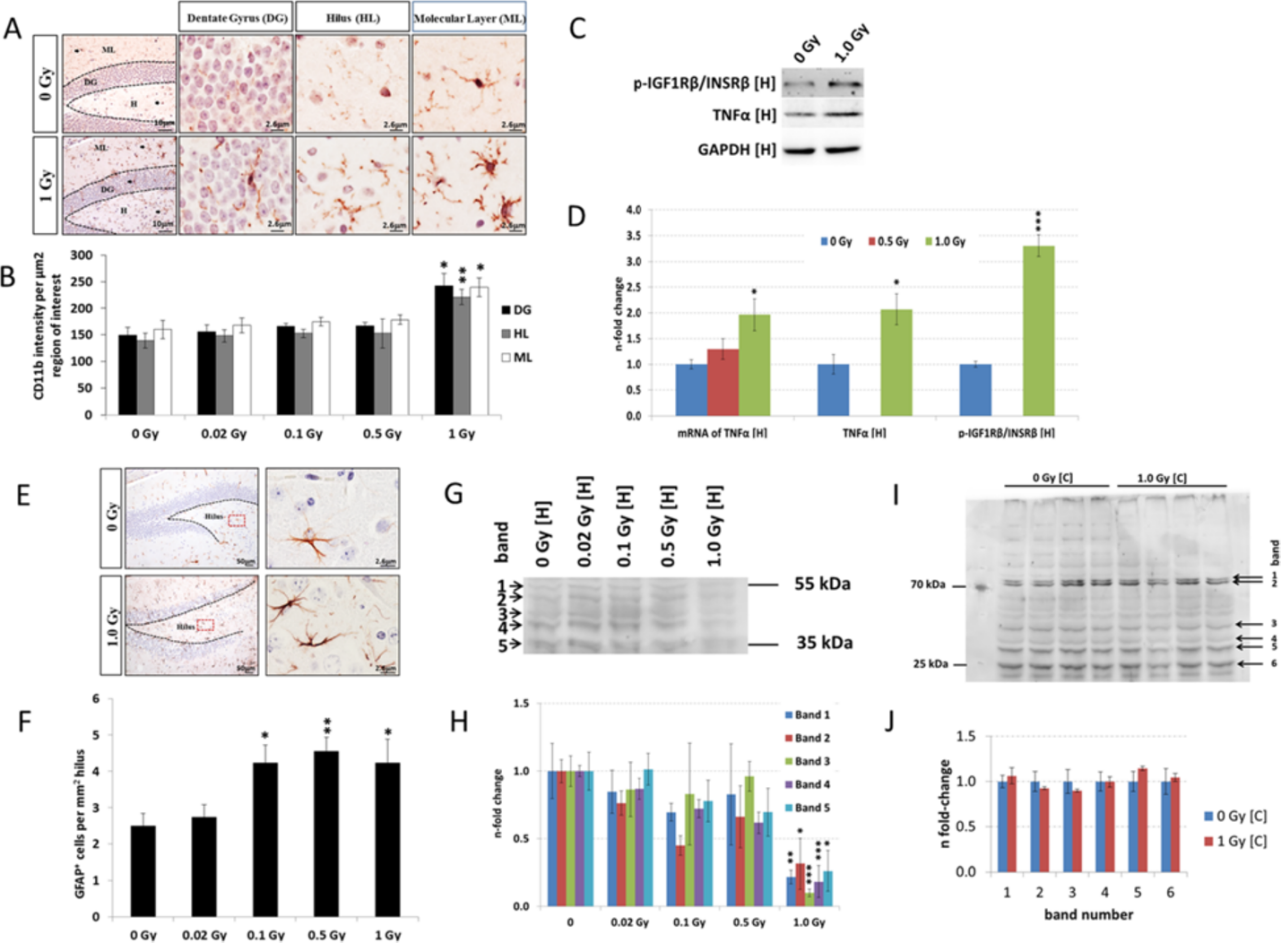
**Evaluation of neuroinflammation, astrogliosis and oxidative stress.** Immunostaining for CD11b showing significantly increased expression in dentate gyrus (DG), molecular layer (ML) and hilus (HL) after irradiation with 1.0 Gy compared to sham-irradiated controls (**A** and **B**) is shown. Gene expression analysis/immunoblots of TNFα, phospho-IGF1Rβ/INSRβ within the hippocampus [H] is shown (**C** and **D**). Figure E and F show immunostaining for GFAP with a significant increase in the number of astrocytes in the hilus after irradiation with 0.1 Gy, 0.5 Gy and 1.0 Gy compared to sham-irradiated controls. Immunoblot of total malondialdehyde (MDA)-tagged proteins within the hippocampus [H] is shown (**G** and **H**). The visualisation of protein bands shows the representative change from the biological replicates; MDA content quantification was performed with 5 bands in the range of 35 kDa to 55 kDa after a total lane normalisation. All data are reported as mean/fold-change ± SEM (n = 5 for immunohistochemistry; n = 4 for immunoblotting against MDA content; n = 3 for gene expression and other immunoblots); *p < 0.05; **p < 0.01; ***p < 0.001 (unpaired Student’s t-test). Immunoblot of total malondialdehyde (MDA)-tagged proteins within the cortex [C] exposed to sham-irradiation and 1.0 Gy **(I)** and quantification of six representative bands in the range of 25 – 70 kDa **(J)** are shown. Statistical analysis was performed with unpaired Student’s t-test. Data are reported as fold-change ± SEM (n = 4). The quantification of the protein bands was done using four biological replicates.

Microglial cells in the irradiated hippocampus showed a more ramified morphology compared to those in sham-irradiated brains, consistent with their activation (Figure 7A). Quantification of the number of CD11b^+^ cells in the three different hippocampal regions [dentate gyrus (DG), molecular layer (ML) and hilus (HL)] showed an increase in CD11b expression at 1.0 Gy (62.2% in DG, 59.13% in HL and 49.6% in ML) (Figure 7B). These changes were accompanied by increased hippocampal transcription and translation of TNFα after 1.0 Gy (Figure 7C - D) and an elevation of 69-82% of GFAP^+^ astrocytes in the hilus at doses ≥ 0.1 Gy (Figure 7E - F).

Further, we detected an increase in the phosphorylation status (active form) of insulin-like growth factor receptor β/insulin receptor β (IGF1Rβ/INSRβ) in the hippocampus at 1.0 Gy (Figure 7C - D). As activation of the IGF-1 pathway may reduce the level of oxidative stress [20] such as deriving from neuroinflammation, we quantified the total malondialdehyde-modified protein content as a surrogate marker for oxidative stress of lipid peroxidation. This demonstrated a significant decrease exclusively at 1.0 Gy in the hippocampus (Figure 7G - H) but not in the cortex (Figure 7I - J).

## Discussion

The long-term consequences of high radiation exposure to the brain regions involved in learning and memory consolidation are not understood. Epidemiological evidence cannot completely exclude that adverse effects may follow a dose as low as that from a single computed tomography (CT) scan of the head [4]. We have therefore investigated the dose–response relationship and biological processes involved in long-term cognitive impairment after irradiation.

### Long-term impairment of cognitive behaviour after irradiation

Genetically out-bred NMRI mice were irradiated at postnatal day ten, representing the developmental stage most sensitive to induced disturbances [21]. Cognitive function was tested by measuring exploratory behaviour after repeated exposure to a new environment [22, 23]. Spontaneous behaviour in a novel home environment depends on mouse ability to integrate the sensoric input into motoric output. This reveals the ability of animals to habituate to a novel home environment and to integrate new information with that previously attained. It can thereby be a measure of cognitive function as habituation is known to be connected to cognitive function [22–24]. In control mice the observed decrease (habituation) in locomotion, rearing and total activity with successive challenges indicates normal cognitive behavior. In irradiated mice, the extent of exploratory behaviour remained sustained until at least 4 months of age in animals exposed to 0.5 and 1.0 Gy indicative for a defective integration of new information into the preexisting network of knowledge. At lower doses the effects were not significant. Although this may indicate a threshold for the damaging effects of radiation, it is equally possible that the robustness of the testing system masks small changes in behaviour at the lower doses.

### Radiation-induced alterations in signalling pathways of synaptogenesis

The alterations detected in signalling pathways promoting synaptic connectivity are consistent with the observed changes in cell kinetics. Ephrin B and ephrin receptor signalling, as well as connecting RhoGDI and Rho family GTPase signalling were changed. Ephrin B signalling is involved in the maintenance of axonal guidance, synaptic plasticity and LTP [25]. These pathways share constituents with the Rac1-Cofilin pathway including Rac1, PAK1/3, Cdc42, LIMK1 and cofilin. The changes imply an overall decrease of the upstream-modulator Rac1 including altered functionality at 0.5 Gy and 1.0 Gy in both hippocampus and cortex. Loss or blockade of Rac1 or Cdc42 reduces neuronal spine morphology and maturation, hippocampal synaptic plasticity and spatial learning [26–28] whereas activation of cerebral RhoGTPases enhances memory for several weeks [29]. A possible mechanism for defects seen in the Rac1-Cofilin cascade may be repression of translation by radiation-induced microRNAs. The miR-132 and miR-134 regulate the Rac1-Cofilin pathway [30, 31]. As LIMK1 is directly targeted by miR-134 [30], the increased levels of miR-134 are in good agreement with the decrease in LIMK1 without corresponding changes in *Limk1* transcripts in both hippocampus and cortex as also shown by others in primary neurons [30]. Moreover, *Limk1*-deficient mice show abnormal spine morphology, reduced dendritic branch size, alteration in hippocampal synaptic plasticity and impaired spatial learning [32]. Further, we observed substantial increase in the level of miR-132, especially in the cortex. Overexpression of miR-132 in the rat perirhinal cortex impairs short-term recognition memory accompanied with attenuated LTD and LTP [33].

Using sequential immunofluorescence, we noted an increase of MAP-2 and PSD-95 protein levels at 1.0 Gy in the hippocampus and DG; these proteins are involved in dendritic spine formation, stability and maturation [18, 19]. The findings are consistent with recently published data showing that PSD-95 levels were increased in neurons of the granule cell layer one month after radiation exposure (1.0 Gy) [10]. Further, the immature filopodia seemed to be highly radiation-sensitive compared to more mature spines [10]. As filopodia are cytoplasmic projections containing actin filaments cross-linked into bundles by Rho family GTPases [34], these data are in good agreement with the alterations seen in the Rac1-Cofilin pathway found in this study.

Overall, we conclude that these effects are indicative for an altered balance of actin polymerisation/-depolymerisation affecting persistently axonal outgrowth and dendritic spine formation. Further investigations on the density and length of mature dendritic spines of hippocampal and cortical neurons may be supportive to elucidate this outcome in more detail.

### Cognitive deficits are accompanied by reduced adult neurogenesis in the hippocampus

The continuous integration of new neurons in the DG contributes to the neural circuit modification involved in memory [35]. This is achieved by proliferation and lineage-specific maturation, with the progression defined by discrete stages of morphology and marker expression. Mature neurons develop from radial glia-like stem cells (type 1, GFAP^+^) and/or non-radial stem cells (type 2b, Sox2^+^) via transient amplifying committed progenitor cells (type 2a, PCNA^+^ and Ki67^+^) to postmitotic immature granule cells (Dcx^+^) and mature neurons (NeuN^+^) [36]. Perturbations in this process of adult hippocampal neurogenesis may result in learning and memory deficits [37]. We showed that irradiation persistently altered cell dynamics within the DG. These changes affect hippocampal circuitry and may explain at least in part the cognitive defects.

Thus, six months after irradiation the DG was depleted in GFAP^+^ type 1 cells, PCNA/Ki67^+^ type 2a cells, and NeuN^+^ mature neurons. Neither Sox2^+^ non-radial stem cells nor Dcx^+^ immature neurons were affected. Overall, we observed a dose-dependency in radiation-induced reductions of hippocampal neurogenesis. These changes are consistent with a continued loss of mature neurons that deplete precursor and stem cell pools.

Alterations in functional small Rho GTPases (Cdc42 and Rac1) may lead to reduced long-term survival of mature neurons [28] as highlighted with our observations of the Rac1-Cofilin pathway. Further, this may be compounded by diversion of precursor cells due to the observed increase in astrocyte differentiation in the hilus. Indeed, radiation has been shown to promote astrocyte-specific differentiation of surviving neural stem cells *in vitro* [38].

### Radiation-induced changes in the CREB-signalling pathway

MiR-212/132 expression is regulated by the CREB transcription factor [39] that is also implicated in dendritic growth [40], neuronal maturation [41], and memory formation [42]. Mice treated with 1.0 Gy showed decreased CREB expression in both hippocampus (~75%) and cortex (~20%). Consistent with the reduced expression of CREB being associated with impaired neural plasticity and rapid forgetting [43] is the observed down-regulation of three plasticity-related genes *c-Fos, Arc and Crem* [44]. The reduced levels of Arc protein may explain in part the observed imbalance in cofilin/phospho-cofilin [45].

Recent studies indicate a regulatory crosstalk between *Arc* and glutamate (AMPA) receptors to modulate synaptic strength whereas inhibition of AMPA/NMDA receptors lead to an increased *Arc* expression via G protein signalling [46].

We noted alterations in the gene expression of NMDA (*Grin2b*) and AMPA (*Gria1,2,3*) receptors but also G-protein coupled glutamate receptors (*Grm3,4,5,7,8*) mainly in hippocampus and, to a lesser extent, in the cortex. Our proteomics analysis in cortex and hippocampus showed increased levels of G-protein nucleotide binding proteins (Gnb’s) (Figure 2C) which are the effector proteins of G-protein coupled glutamate receptor indicative for a potential G-protein coupled receptor activation. Moreover, we observed gene expression alterations of protein kinases and calcium-dependent phosphatases that modulate synaptic plasticity through AMPA and NMDA receptors [47]. Further experiments using electrophysiological or electroencephalographical methods are important to clarify the molecular changes seen in the CREB and LTP/LTD-dependent signalling.

### Induction of neuroinflammation in hippocampus

Brain plasticity, neuronal networks and cognitive performance may all be influenced by crosstalk with the immune system [48]. Consistent with a role of inflammatory processes in the radiation-induced cognitive impairment were the observed increase in microglia, raised levels of TNF-alpha, and sustained phosphorylation of the IGF/INS receptors in the radiation-treated hippocampus (1.0 Gy). Prevention of a radiation-induced inflammation by indomethacin in rodents restricted cognitive impairment [49]. Similarly, insulin-like growth factor 1 (IGF1)-dependent signalling was shown to modulate cognitive behaviour [50]. Further, a link between inflammation, neuronal dysfunction and defective insulin signalling in Alzheimer’s disease has been demonstrated [51]. Insulin-like growth factor- and insulin-receptors are upstream regulators of PI3K/Akt and Rac1-Cofilin pathway [52]. As activation of the PI3K/Akt pathway lowers lipid peroxidation as shown in primary cortical neuronal cultures [53] and mouse hippocampal neurons (HT22 cells) [54], these data agreed with the observed significant reduction of lipid peroxidation in hippocampus (1.0 Gy).

Further investigations of irradiated mice treated with chemical agents or genetic manipulation that inhibit microglial activation may lead to a better understanding of the long-term role of neuroinflammation in regard to cognitive function after neonatal irradiation.

## Conclusions

**Figure 8:**
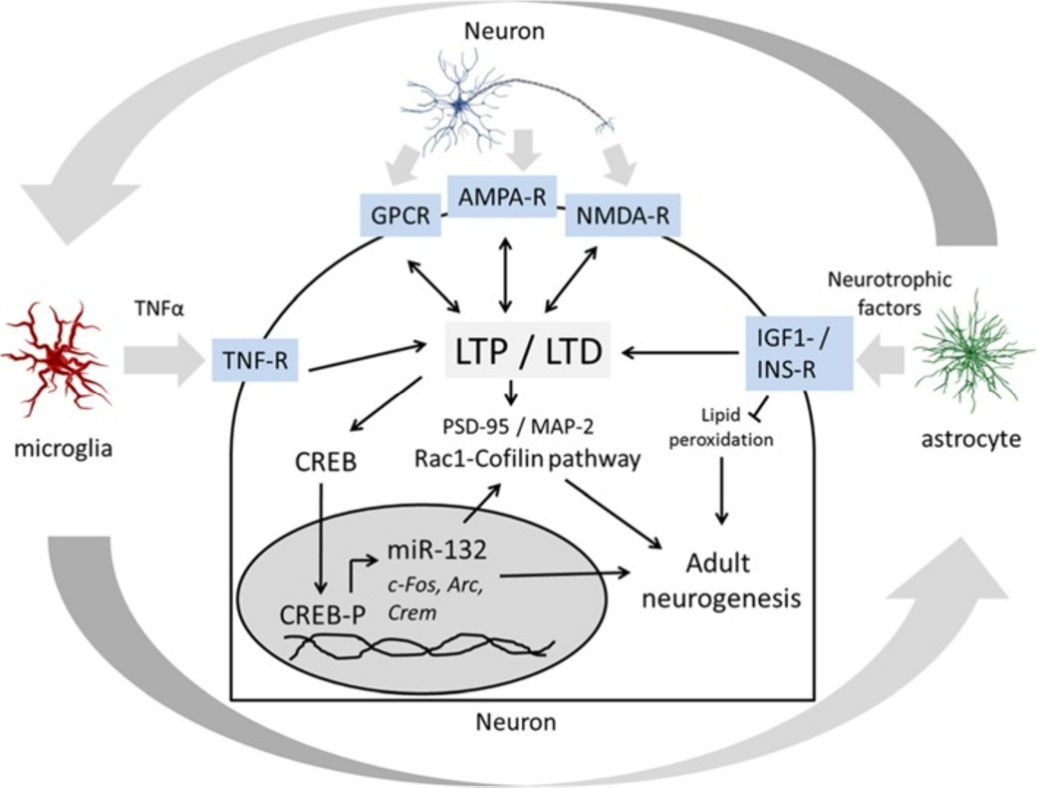
**Potential mechanism of radiation-induced persistent learning and memory deficit.** Schematic presentation of long-term effects of ionising radiation on the brain combining all presented data from 1.0 Gy-exposed hippocampus is shown. Microglia and astrocytes regulate neuronal activity by influencing cytokines (TNFα) or neurotrophic factors. This results in changes in the receptor profile of G protein coupled receptors (GPCR’s) and AMPA and NMDA receptors which are important for steady state signal transmission from neuron to neuron. In turn, this affects long-term potentiation/long-term depression (LTP/LTD) and leads to alterations in synaptic morphology (actin reorganisation via Rac1-Cofilin-pathway, changes in synaptic scaffold proteins such as PSD-95/MAP-2) and synaptic plasticity (CREB pathway). Phosphorylated CREB (CREB-P) regulates the expression of miR-132 and immediate early genes such as c-Fos, Arc and Crem affecting both synaptic morphology and adult neurogenesis. The connecting arrows are based on the literature mentioned in the discussion; genetic, chemical or pharmacological manipulations have to be performed to ascertain these links in detail.

Taken together, we have correlated persistent changes in cognitive performance with marked defects in adult hippocampal neurogenesis, increased neuroinflammation, and alterations in synaptic plasticity in the hippocampus and cortex. The complex radiation-induced long-term consequences cannot be explained by single cell type alterations but rather by dynamic interaction of multiple cell types including neurons, microglia and astrocytes (Figure 8). A better understanding of the mechanisms of cognitive and neurological dysfunction at low and moderate radiation doses is of critical importance in minimising radiation-associated health risks and in planning radiation protection policies and regulations.

## Methods

### Irradiation of animals and transport to site of analysis

All experiments were carried out in accordance with the European Communities Council Directive of 24 November 1986 (86/609/EEC), after approval from the local ethical committees (Uppsala University and the Agricultural Research Council) and by the Swedish Committee for Ethical Experiments on Laboratory Animals. Male NMRI mice (Charles River, Germany) were exposed to a single dose of total body gamma irradiation of 0 (sham), 0.02, 0.1, 0.5 and 1.0 Gy on PND 10 (^60^Co source, 0.025 Gy/min) (The Svedberg laboratory, Uppsala University) without anaesthesia. Neonates were not restrained during irradiation and could freely move during treatment. Dose verification was done with an ionisation chamber (Markus chamber type 23343, PTW-Freiburg). Three litters were used within each irradiation group to minimise inter-litter effects. Mice were kept at Uppsala University for behavioural testing until age of 5 months and were sent then to Germany receiving a routine treatment for intestinal parasites with Ivomac (Merial, 0.03 mg/mouse, over 1 week) [55] and Italy. At age of 6 months (histological analysis) or 7 months (all other analyses), animals were sacrificed. Animals were kept at all times under normal housing conditions [8]. Animals were sacrificed via cervical dislocation (for immunofluorescence and immunohistochemistry) and CO_2_ (other experiments).

### Behavioural testing

Spontaneous exploratory behaviour in a novel home environment was observed in male mice as previously described using an automated activity monitor [8, 56]. 12 animals taken from a minimum of three different litters were used per exposure group for behavioural testing. The locomotion, rearing, and total activity data was determined over three consecutive 20-min periods. Spontaneous behaviour tests were submitted to a split-plot ANOVA design [57] by using software package SAS 9.1. Pair-wise testing between the different doses of gamma-radiation and sham-irradiated controls was done with Duncan’s MRT test.

### Mass spectrometry-based proteome analysis

Brains were removed to ice-cold phosphate-buffered saline (PBS), rinsed and stereomicroscopically dissected under cold conditions. Hippocampi and cortices without meninges from each hemisphere were separately collected, gently rinsed in ice-cold PBS and snap-frozen in liquid nitrogen. Samples were stored at -20°C for further analysis. Samples were processed for proteomic analysis as previously described [58]. Protein extracts from hippocampi and cortices were labelled with isotope coded protein label (ICPL) triplex reagent (SERVA Electrophoresis GmbH, Germany) according to the manufacturer’s instructions. Individual protein lysates (50 μg in 20 μl of 6 M guanidine hydrochloride from each cortex and hippocampus sample) were reduced, alkylated and labelled with the respective ICPL-reagent as follows: sham-irradiated sample with ICPL-0, 0.02 Gy or 0.1 Gy treated sample with ICPL-4 and 0.5 Gy or 1.0 Gy treated samples with ICPL-6. The labelled samples from five cortices and four hippocampi from each irradiation group were combined (sham-0.02Gy-0.5Gy and sham-0.1Gy-1.0Gy) followed by overnight precipitation with 80% acetone at -20°C to purify the labelled protein content. Biological replicates included animals from three different litters. Precipitates were suspended in Laemmli buffer and separated by 12% SDS-polyacrylamide gel electrophoresis followed by Coomassie blue staining. Gel lanes were horizontally cut into six equal slices, destained and trypsinised overnight as described recently [59]. Peptides were extracted and acidified with 1% formic acid and analysed via mass spectrometry.

LC-MS/MS analysis was performed on a LTQ-Orbitrap XL (Thermo Fisher, Germany) as described previously [60]. Briefly, the gel slice fractionated samples were automatically injected and loaded onto the trap column and after 5 min peptides were eluted and separated on the analytical column by reversed phase chromatography operated on a nano-HPLC (Ultimate 3000, Dionex, Germany) with a nonlinear 170 min gradient using 35% acetonitrile in 0.1% formic acid in water (A) and 0.1% formic acid in 98% acetonitrile (B) at a flow rate of 300 nl/min. The gradient settings were: 5–140 min: 14.5-90% A, 140–145 min: 90% A - 95% B, 145–150 min: 95% B and equilibration for 15 min to starting conditions. From the MS pre-scan, the 10 most abundant peptide ions were selected for fragmentation in the linear ion trap if they exceeded an intensity of at least 200 counts and were at least doubly charged. During fragment analysis, a high-resolution (60,000 full-width half maximum) MS spectrum was acquired in the Orbitrap with a mass range from 200 to 1500 Da.

MS/MS spectra were searched against the ENSEMBL mouse database via MASCOT software with a mass error and fragment tolerance of 10 ppm and 0.6 Da, including not more than one missed cleavage. Fixed and variable modifications were carbamidomethylation of cysteine and ICPL-0, ICPL-4 and ICPL-6 for lysine. Proteins were identified and quantified based on the ICPL pairs using the Proteome Discoverer software (Version 1.3 – Thermo Fisher, Germany). To ensure that only high-confident peptides were used for protein quantification, we applied the MASCOT percolator algorithm [61] as used previously [62, 63]. The q value of the percolator algorithm was set to 0.01 representing strict peptide ranking. Thus, only the best ranked peptides were used. Further, these peptides were filtered against a Decoy database resulting in a false discovery rate (FDR) of each LC-MS-run; the significance threshold of the FDR was set to 0.01 to ensure that only highly confident peptides were used for protein quantification. Proteins from each LC-MS-run were normalised against the median of all quantifiable proteins. Proteins were considered significantly deregulated if they fulfilled the following criteria: (i) identified by at least two unique peptides in three out of the four hippocampal biological replicates and four out of the five cortical biological replicates, (ii) quantified with a ICPL-4/ICPL-0 and ICPL-6/ICPL-0 variability of ≤ 30% and (iii) had a fold-change of ≥ 1.3 or ≤ -1.3. The threshold of ±1.3 is based on the average experimental technical variance of the multiple analysis of hippocampal and cortical technical replicates (13.8%) (Additional file 1: Table S1). Further, it enables the unbiased confident quantification of deregulated proteins regardless of applied radiation dose (Additional file 1: Table S2).

### Data deposition of proteomics experiments

The raw-files of the obtained MS-MS spectra can be found under http://storedb.org/project_details.php?projectid=29 with the ProjectID 29.

### Analysis of protein classes and affected signalling pathways

Deregulated proteins were categorised into protein classes using the PANTHER classification system software (http://www.pantherdb.org) and the general annotation from UniProt (http://uniprot.org). The analysis of the affected signalling pathways for all deregulated proteins was performed with INGENUITY Pathway Analysis (IPA) (http://www.ingenuity.com) applying databases of experimental and predictive origin.

### Immunoblotting analysis

Hippocampal and cortical protein extracts (15 μg) were separated on 12% SDS polyacrylamide gels and were blotted to nitrocellulose membranes (GE Healthcare, Germany) via BIO-RAD criterion™ Blotter system at 100 V for 2 h. Membranes were blocked with Roti^R^-Block solution (Roth, Germany), washed and incubated overnight at 4°C with primary antibody dilutions as indicated by the manufacturer (GAPDH – sc-47724 Santa Cruz, Germany; CDC42 – ab106374 Abcam, Germany; Rac1 – ab33186 Abcam, Germany; p-PAK1 – 2601 Cell Signaling, Germany; Fascin – ab74487 Abcam, Germany; Cofilin – 3312 Cell Signaling, Germany; Rho-GDIα – ab108977 Epitomics, Germany; p-LIMK1/2 – sc-28409-R Santa Cruz, Germany; p-Cofilin – 3311 Cell Signalling, Germany; LIMK1 – 3842 Cell Signaling, Germany; p-Rho-GDIα – sc-33047 Santa Cruz, Germany; TNFα – 3707 Cell Signaling, Germany; p-IGF1Rβ/INSRβ – 3021 Cell Signalling, Germany; Arc – ab118929 Abcam, Germany; c-Fos – sc-52 Santa Cruz, Germany; p-CREB – 9191 Cell Signaling, Germany; CREB – 4820 Cell Signaling, Germany). After a washing step, blots were incubated with appropriate horseradish peroxidase-conjugated secondary antibodies in 8% milk for 1 h at room temperature and developed using the ECL system (GE Healthcare, Germany) using standard procedures. GAPDH was not significantly deregulated either at the mRNA or protein level in any sample and was therefore used as the loading control. Each irradiated group was run on separate immunoblots with corresponding sham-irradiated control samples under identical conditions on the same day. Immunoblots were only considered for quantification (TotalLab TL100 software; http://www.totallab.com) if the ratios between control samples and endogenous GAPDH were similar after software-suggested background correction. Three biological replicates were used for statistical analysis (unpaired Student’s t-test) with a significance threshold of 0.05.

Detection of global lipid peroxidation was done via quantification of malondialdehyde-tagged proteins using a primary antibody against malondialdehyde (MDA11-S Alpha Diagnostic, USA). A total of four immunoblots (50 μg protein lysate per lane) were run on the same day under identical conditions using the corresponding three control samples on each blot and four biological samples from each irradiation group. Immunoblots were considered for quantification if (i) the pattern and intensity of lanes stained with Ponceau S were equal and (ii) total lane intensity of malondialdehyde-tagged proteins was similar. Five bands per lane in the range of 35 – 55 kDa were selected as representative indicators of global lipid peroxidation and were used for further quantification. Each band was normalised against the total lane intensity and fold-changes were calculated from each blot separately. Three and four biological replicates of control and irradiated samples were used for statistical analysis, respectively. Data analysis was done from two independent technical experiments (Student’s t-test, unpaired).

### Transcriptomics and microRNA analysis

Total RNA from frozen hippocampi and cortices was isolated and purified using the mirVana™ Isolation Kit (Ambion, Germany) according to the manufacturer’s protocol. For both microRNA and gene expression studies the OD ratio of 260 nm / 280 nm from RNA lysates was estimated using a Nanodrop spectrophotometer. This ratio reflecting the RNA quality ranged between 2.0 and 2.1. Obtained RIN values ranged between 8.6 and 9.0 (TapeStation, Lab901). Eluates were stored at -20°C until further analysis.

Hippocampal and cortical RNA isolates (100 ng) were used to quantify the gene expression of 84 genes [“synaptic plasticity” (sham, 0.5 Gy, 1.0 Gy), “PI3k/Akt signalling pathway” (sham, 0.5 Gy, 1.0 Gy) and “circadian rhythm” (sham, 1.0 Gy) (RT^2^ Profiler PCR array – Qiagen, Germany)]. The assays were performed following manufacturer’s instructions on a StepOnePlus (Applied Biosystems, Germany) using RT^2^SYBR Green Mastermix. For pathway-focused transcriptomics, the relative expression of each mRNA was normalised against the median of all 84 target genes using the equation 2^-ΔΔCt^, where ΔΔCt = ΔCt_irradiated_ – ΔCt_sham_ and ΔCt = Ct_target-mRNA_ – Ct_median-of-84-target-genes_. Three biological replicates from three different litters were used within each dose group. Gene expression changes were considered significant if they reached a p-value of ≤ 0.05 and had a fold-change of ≥ 1.2 or ≤ -1.2. The threshold of ± 1.2 is based on the average experimental technical variance (8.4%) and biological variance (6.9%) of a set of 14 overlapping target genes (Additional file 1: Table S3). Results from overlapping gene targets were only regarded as significantly deregulated if they (i) were consistently up- or down-regulated, (ii) had overlapping confidence intervals and (ii) were consistently significantly changed.

Expression of single miRNAs using the QuantiTect Reverse Transcription Kit (Qiagen, Germany) and single miRNAs using the TaqMan Single MicroRNA Assay (Applied Biosystems, Germany) were performed following manufacturer’s protocol on a StepOnePlus device (Applied Biosystems, Germany) using Taqman-primers. For single mRNA and miRNA quantification, following Taqman-primers were used: mmu-miR-132 (ID000457), mmu-miR-134 (ID001186), mmu-miR-212 (ID002551), snoRNA135 (ID001239), *Tnfα* (Mm00443260_g1), *Gapdh* (Mm99999915_g1) and *Limk1* (Mm01196310_m1) - all from Life Technologies, Germany. Potential contamination with genomic DNA was excluded using the same conditions but without reverse transcriptase. Expression levels of miRNA and mRNA were calculated based on the 2^-ΔΔCt^ method with normalisation against endogenous snoRNA135 [64] and *Gapdh*, respectively. Changes were considered significant if they reached a p-value of ≤ 0.05 (unpaired Student’s t-test, n = 3).

### Immunohistochemistry and immunofluorescence

Formalin fixed and paraffin embedded tissues were prepared using standard techniques [65]. For immunohistochemistry, one μm thick single (stainings of NeuN and Ki67) and 4 μm thick serial (stainings of Caspase-3, GFAP, Sox2, Dcx, CD11b, PCNA and γH2AX) sagittal whole brain sections were dewaxed, rehydrated and heated in citrate buffer for 30 minutes. Quenching of endogenous peroxidase was performed with 3% H_2_O_2_ in methanol. Brain sections were incubated with primary antibody dilutions as indicated by the manufacturer. Following antibodies were used for immunohistochemistry: Caspase-3 (9664 – Cell Signaling, Germany), GFAP (Z0334 – Dako, Germany), Sox2 (ab97959 – Abcam, Germany), Dcx (ab97959 – Abcam, Germany), CD11b (ab1211 – Abcam, Germany), PCNA (NA03 mAB-1 – Calbiochem, Germany), γH2AX (05636 – Upstate Biotechnologies Inc., USA), Ki67 (ab15580 – Abcam, Germany) and NeuN (MAB377 – Millipore, Germany). Immunohistochemical analysis was done using polyclonal antibodies against Sox2, Dcx and CD11b. Complexes were visualised using a rabbit biotinylated-conjugated secondary antibody; after incubation with avidin-biotin immunoperoxidase staining, the antibody-antigen complexes were visualised with Vector NovaRED Substrate Kit (Vector Laboratories Inc., CA, USA) according to manufacturer’s instructions. Antibody–antigen complexes of polyclonal GFAP or caspase-3 were visualised using horseradish peroxidase-conjugated secondary antibody and the DAB chromogen system (Dako North American Inc, CA, USA). Immunohistochemical analysis of monoclonal antibody against γ-H2AX and PCNA was performed using the HistoMouse MAX Kit (Invitrogen Corporation, CA). The staining against NeuN was perfomed using the MoMap Kit (760–137 - Ventana, Germany), according to manufacturer’s instructions. Antibody-antigen complexes of Ki67 and NeuN were visualised using soluble immune complex of biotinylated secondary and mouse primary antibody (MoMap Kit 760–137 - Ventana, Germany) or rabbit biotinylated-conjugated secondary antibody. Subsequently, slides were incubated with avidin-biotin horseradish immunoperoxidase and were visualised with diaminobenzidine (DAB) (Sigma Aldrich, Germany).

The number of positive cells for GFAP or Sox2 in the subgranular zone (SGZ) was determined and expressed as a fraction of labelled cells out of the total number of granule neurons in the DG. To quantify PCNA or Dcx, the number of positive cells in the SGZ was expressed per μm^2^ of DG. Immunohistochemical staining for NeuN was performed to assess the neuronal density in the granule cell layer of the DG. Counting was carried out in a rectangular field of 4000 μm^2^ in the supra- and infrapyramidal blade and in the crest area of the DG. The number of positive cells in each of the areas was recorded separately followed by statistical analysis of the mean from three biological replicates. For quantification of microglia in brain sections immunostaining for CD11b was imaged by HistoFAXS (TissueGnostic, Austria). Regions of interest were selected in the molecular layer, DG and hilus and analysed with HistoQuest (TissueGnostics, Austria) using automatic colour separation and quantification. Quantitative analysis of astroglial cells (labelled by GFAP antibody) was performed by counting positive cells in the hippocampus (H) area (GFAP cells/H area [μm^2^]). Both DG and H area were measured by imaging software NIS-Elements BR4.00.05 (Nikon, Instruments Europe B.V., I) after tracing the DG / H outline.

All images were analysed using identical software settings. Statistical analysis (Student’s t-test, unpaired) was performed with six biological replicates for stainings of Caspase-3, GFAP, Sox2, Dcx, CD11b, PCNA and γH2AX and three biological replicates for stainings of Ki67 and at least three biological replicates for NeuN. Differences were considered to be significant when p-values were < 0.05 using unpaired Student’s t-test.

For immunofluorescence, one μm thick brain sagittal sections were dewaxed, rehydrated and heated in citrate buffer followed by auto-fluorescence block (0.1% sudan black in 70% ethanol). After a goat serum block, slides were overnight incubated with rabbit anti-mouse primary antibody against MAP-2 (ab32454 – Abcam, Germany) followed by goat anti-rabbit Cy3-Fab-fragment IgG secondary antibody (111-167-003 - Jackson ImmunoResearch, UK) after manufacturer’s instructions. Subsequently, the slides were washed in PBS and overnight incubated with rabbit anti-mouse primary antibody against PSD-95 (ab18258 – Abcam, Germany) followed by goat anti-rabbit Alexa-fluor IgG secondary antibody (111-545-144 - Jackson ImmunoResearch, UK) after manufacturer’s indications. Subsequently, the slides were nuclear stained with Hoechst and mounted with antifade fluorescence mounting media. Sample processing was done under identical conditions on the same day. All images were analysed using identical software settings. The MAP-2 / PSD-95 intensity in the region of interest was normalised against the Hoechst intensity within this region. Three biological replicates were used in all cases. Statistical significance was calculated with unpaired Student’s t-test.

## Electronic supplementary material

Additional file 1: Table S1: Calculation of the average technical variance of proteomics experiments via repetitive measurement of technical replicates. **Table S2.** Evaluation of biological depth of proteomics in brain samples in regard to applied ionising radiation dose. **Table S3.** Calculation of the average technical and biological variance of transcriptomics experiments via overlapping target genes. **Table S4.** List of quantifiable cortical and hippocampal proteins at different radiation doses using proteome analysis. **Table S5**. List of genes quantified by the arrays “PI3k/Akt signalling pathway”, “synaptic plasticity” and “circadian rhythm”. **Table S6.** Protein list of significantly changed cortical and hippocampal proteins found in mass spectrometry-based ICPL approach. (XLS 2 MB)

## Abbreviations

ANOVA: Analysis of variance
AMPA: α-amino-3-hydroxy-5-methyl-4-isoxazolepropionic acid
C: Cortex
CNS: Central nervous system
CR: Crest area
CT: Computed tomography
DG: Dentate gyrus
DAB: Diaminobenzidine
FDR: False discovery rate
GO: Gene ontology
H: Hippocampus
HL: Hilus
HPLC: High pressure liquid chromatography
ICPL: Isotope coded protein label
IB: Infrapyramidal-blade
IEG: Immediate-early response gene
IPA: Ingenuity pathway analysis
LC-MS/MS: Liquid chromatography tandem mass spectrometry
LTP: Long term potentiation
LTD: Long term depression
MDA: Malondialdehyde
miRNA: microRNA
ML: Molecular layer
NMDA: N-methyl-D-aspartate
PND10: Postnatal day 10
PBS: Phosphate buffered saline
SB: Suprapyramidal-blade
SDS: Sodium dodecyl sulphate
SEM: Standard error of the mean
SGZ: Subgranular zone.
